# Maternal Tobacco and Alcohol Use in Relation to Child Malnutrition in Gauteng, South Africa: A Retrospective Analysis

**DOI:** 10.3390/children8020133

**Published:** 2021-02-11

**Authors:** Perpetua Modjadji, Mpinane Pitso

**Affiliations:** School of Health Care Sciences, Department of Public Health, Sefako Makgatho Health Sciences University, 1 Molotlegi Street, Ga-Rankuwa, Pretoria 0208, South Africa; Mpinane.makhoathi@smu.ac.za

**Keywords:** tobacco and alcohol use, mothers, malnutrition, children, township setting, South Africa

## Abstract

Tobacco and alcohol use among mothers is associated with numerous adverse consequences for affected offspring, including poor growth and development. This study determined the association between maternal tobacco and alcohol use, and malnutrition, among infants aged ≤ 12 months (*n* = 300), in selected health facilities situated in Gauteng, South Africa. Data on alcohol and tobacco use were collected using a validated questionnaire, in addition to mothers’ socio-demographic characteristics and obstetric history. Stunting (low height/length-for-age), underweight (low weight-for-age) and thinness (low body mass index-for-age) were calculated using z-scores based on the World Health Organization child growth standards. The association of tobacco and alcohol use with stunting, underweight and thinness was analysed using logistic regression analysis. The results showed a mean age of 29 years (24.0; 35.0) for mothers and 7.6 ± 3 months for infants, and over half of the mothers were unemployed (63%). Approximately 18.7% of mothers had used tobacco and 3% had used alcohol during pregnancy. The prevalence of current tobacco and alcohol use among mothers were estimated at 14.3% and 49.7%, respectively, and almost three-quarters (67.3%) of them were still breastfeeding during the study period. Stunting (55%) was the most prevalent malnutrition indicator among infants, while underweight was 41.7%, and thinness was 22%. Current tobacco use was associated with increased odds of being thin [OR = 2.40, 95% CI: 1.09–5.45), and after adjusting for confounders, current alcohol use was associated with the likelihood of being underweight [AOR = 1.96, 95% CI: 1.06–3.63] among infants. Future prospective cohort studies that examine growth patterns among infants who are exposed to maternal tobacco and alcohol use from the intrauterine life to infancy are necessary to inform, partly, the public health programmes, to reduce malnutrition among children.

## 1. Introduction

Tobacco and alcohol are the psychosocial factors contributing to public health issues in low and middle-income countries (LMICs) [1,2,3,4]. In Africa, the prevalence of alcohol use during pregnancy ranges from 4.30% to 59.28%, and varies within and across countries, while in Sub-Saharan Africa (SSA), the prevalence has been estimated between 4.30% and 59.28% [5]. These variations have been reported to originate from factors, such as maternal drinking behaviors, environmental, policy, and cultural differences [6]. Additionally, several studies conducted from the African context have reported high rates of alcohol consumption during pregnancy, with implications on having partners who consume alcohol, peer pressure, and lack of sufficient information about the adverse health effect [7,8,9]. In addition to alcohol use, tobacco use (both smoked and smokeless) is one of the public health threats [10,11,12], especially among pregnant women and their children [12,13]. The overall use of tobacco among pregnant women in SSA has been estimated at 2%, but some countries, such as Burundi (4.2%), Lesotho (5.4%), Madagascar (11%), Namibia (4.4%), and Sierra Leone (4.8%) have reported higher prevalence [12]. Predictors of tobacco use, such as age, religion, and wealth index have been reported among pregnant women in SSA [12].

In South Africa, use of tobacco and alcohol among women of reproductive age has been documented with varying rates between the provinces and different racial groups [4,14], reflecting the differences in sociocultural and demographic determinants [5,15,16]. In the nine provinces the country has, Western Cape Province has reported approximately over 50% of alcohol use [17,18]. High prevalence of maternal alcohol use during pregnancy has also been reported in other provinces like Free State and Northern Cape (6.1–7.3%) [19], while relatively low prevalence of alcohol use during pregnancy has been reported in Limpopo Province (0.07%) [20]. As early as 2000, May et al. reported high prevalence of tobacco use during pregnancy among mothers of children without foetal syndrome (FASD) (46.3%) and mothers of children with foetal alcohol syndrome (FAS) (87.9%) in the Western Cape Province [17]. Substantial discrepancies in tobacco use between the provinces and different racial groups in South Africa have been reported, which suggest variances in the contributing factors of tobacco use, such as socio-cultural and demographic factors [16].

The relationship between alcohol use and nutrient status has been studied in numerous epidemiologic studies, and the quality of diet is usually compromised for individuals with high alcohol quantities and frequency [21]. Studies conducted in the Western Cape Province have reported micronutrients deficiencies among mothers of children who suffered from FASD [22,23]. Literature further documents that maternal body mass index (BMI), together with the history of alcohol use, impact on the multifaceted interaction between a mother and the foetus [24]. Additionally, breastfeeding has always been considered as the best method for promoting infant growth and health [25]. However, the belief that alcohol use during breastfeeding has harmful consequences on child development has been long held, although there is a paucity on empirical evidence [25]. Use of alcohol during breastfeeding may deprive newborns to insufficient nutrients through breastmilk and expose them to the teratogen effect of alcohol [26]. In some parts of South Africa, researchers have reported that alcohol use during the period of breastfeeding significantly compromises child development [27].

Maternal use of tobacco and alcohol during pregnancy, as well as, in the period prior to conception, and thereafter, determine the health outcomes of children [28]. Alcohol use during pregnancy is associated with low birth weight, preterm delivery, and foetal alcohol syndrome [29,30,31]. This is because alcohol during pregnancy exposes a child to inadequate essential nutrients through a compromised nutritional status of the mother [23]. On the other hand, the risks associated with tobacco smoking during pregnancy for both mother and child have been established [32]. Pregnancy complications, such as preeclampsia and poor foetal outcomes (i.e., low birthweight and premature birth), are well established [33,34]. As a result, the combined use of tobacco and alcohol is associated with poor infant outcomes, including poor growth [4]. It is also worth noting that second hand smoke (SHS), which usually occurs at home, work, and hospitality venues [35], affects children and women in their daily lives [36], and brings about almost the same adverse health outcomes as active smoking [37].

Malnutrition is considered as a set of symptoms of developmental impairment, especially growth failure, among other abnormalities [16]. Particularly undernutrition, a type of malnutrition characterised by undernourishment, poor absorption and use of biological use of nutrients consumed, constitutes underweight (low weight for one’s age), stunting (too short for one’s age), wasting/thinness (thin for one’s height), and micronutrients deficiencies status [17]. A foetus exposed to alcohol might have suboptimal outcomes, especially physical abnormalities, due to the compromised nutritional status of mothers because of inadequate essential nutrients [24]. South Africa has recorded 32% of stunting among children under 2 years and underweight (5.9%) and wasting/thinness (2.3%) [1], although, prevalence as high as 58% for stunting and 13% for wasting have been reported among children in selected health facilities situated in the Western Cape, Free State, and Northern Cape Provinces [38]. The differences in the prevalence of underweight, stunting and wasting/thinness may occur in multi-ethnic population groups, which could be due to the genetic, environmental, and psychosocial factors [39,40,41,42].

While FASD is well studied in South Africa, especially in the Western Cape Province [18,23,27,43], there is a paucity of data on the association of child malnutrition/infant growth, and maternal tobacco and alcohol use in South Africa. Most of the local studies have studied child malnutrition without taking into consideration the use of alcohol and tobacco [44,45,46,47], except for minimal research, which has an observable fact [41]. It is well documented that children in developing countries, such as South Africa, face several adversities that affect their growth, which arise as early as intra-uterine life [41]. Some of the adversities revolve around substance abuse, mainly alcohol and tobacco [41]. A substantial proportion of women of reproductive age in South Africa have been reported to engage in alcohol and tobacco use, even during pregnancy [19,43], while child malnutrition remains a multifaceted public health problem in South Africa [47,48]. Therefore, this study aimed to determine the association of maternal tobacco and alcohol use with malnutrition indicators among infants in a number of health facilities in Gauteng, South Africa.

## 2. Methods

### 2.1. Study Design

A cross sectional study using retrospective analysis was conducted between September and November 2019. Primary health care (PHC) facilities were used and considered relevant to access mothers who attend health services.

### 2.2. Study Setting

Tshwane Health District, situated in Gauteng Province, was used as a study area. The district has a total population of 2,708,702 people and is made up of several townships [49]. Townships in a South African context are under-developed residential areas that during apartheid in South Africa were reserved for Africans, Coloureds, and Indians to live [50]. Eersterust, a township situated in one of the seven sub-districts of Tshwane Health District, was chosen as a suitable study setting based on anecdotal evidence that the township is one of the main places faced with challenges of substance abuse, especially alcohol. All the three public PHC facilities in Eersterust serving the community were used in the study.

### 2.3. Study Participants

The study included women of reproductive age who had given birth during the preceding 4 weeks to 12 months and who were attending mother and child health services at the three selected PHC facilities in City of Tshwane. Biological mothers participated with their children aged between 4 weeks and 12 months. Infants aged lesser than 4 weeks, older than 12 months, having noticeable physical disability, and reported to be sick by the mothers at the time of the study were excluded.

### 2.4. Sample Size and Sampling Procedure

Raosoft sample size calculator [51] was used to calculate a minimum sample of 280, considering the population size of approximately 1200 mothers, who had attended the service in the past 3 months prior to the current study, based on the information obtained from the managers in charge of the facilities. The sample was buffered with 10% to avoid the size being compromised by non-response, and a final sample size of 308 was obtained. Failure to obtain a random sampling, convenience sampling, applicable in clinical research [52], was used to select mothers who agreed to participate in the study.

### 2.5. Data Collection

#### 2.5.1. Socio-Demographic and Obstetric History of Mothers

Research assistants collected data on the socio-demography and obstetric history, using a validated questionnaire. The questionnaire was adapted from literature [44,45] and validated through face and content validity, translation, and a pilot study. During the pilot study, research assistants were trained on data collection, which includes administration of a questionnaire. Further questions on maternal alcohol use were also adapted from literature [19]. Additionally, questions on maternal tobacco were added to the developed questionnaire. Maternal alcohol use during pregnancy and the current period was measured through the following questions: (1) during pregnancy, did you ever have a drink containing alcohol? (yes or no) and (2) Are you currently drinking alcohol? (yes or no). Maternal tobacco use during pregnancy and the current period were assessed using the following questions: (1) during pregnancy, did you ever use tobacco? (yes or no) and (2) Are you currently using tobacco? (yes or no) [19].

#### 2.5.2. Anthropometric Measurements of Children and Mothers

Smart D-quip electronic scale and a non-stretchable tape measure were used to measure infants’ weights (W) and recumbent length (L), respectively, based on the standard procedures of WHO [53]. WHO Anthro Software was used to generate the z-scores: length/height-for-age (HAZ), weight-for-age (WAZ), and body mass index-for-age (BAZ). Stunting (low HAZ) and underweight (low WAZ) were defined at z-scores between −3 SD to −2 SD. Thinness (low BAZ) was defined at z-scores < −2 SD [53]. BMI-for-age (BAZ) scores between +1 SD and +2 SD indicated possible risk of overweight, while overweight was at BAZ > +2 SD and obesity at BAZ > +3 SD. Research assistants measured anthropometry.

Maternal weights and heights were measured using a calibrated smart D-quip electronic scale and a stadiometer, respectively. Three measurements were taken according to WHO procedures and the average measurements for weight (W) and height (H) were recorded. A BMI between 18.5 to 24.99 kg/m^2^ was considered normal, while underweight, overweight, and obesity were defined at BMI <18.5 kg/m^2^, between 25 to 29.99 kg/m^2^, and ≥30 kg/m^2^, respectively [54,55].

### 2.6. Statistical Analysis

STATA 14 (StataCorp. 2015. Stata Statistical Software:Release 14. College Station, TX, USA) was used to analyse data. Tests for normality (i.e., Skewness–Kurtosis) determined the distribution of continuous data for mothers and for infants. The medians of malnutrition indicators among children were compared by sex using Mann–Whitney test. The results are presented as median [Interquartile range (IQR)]. Logistic regression analysis was used to determine the association of tobacco and alcohol use (in the current period and during pregnancy), with malnutrition indicators. The independent strength of the association was determined through the adjusted odds ratios (AOR) with a 95% confidence interval (CI). We ran model for the overall sample of children’s nutritional indicators (i.e., HAZ, WAZ and BAZ) for the pregnancy period and the current period, which excluded confounders, such as child’s sex, maternal age, BMI, marital status, education level, employment status, family size, monthly income, house type, and access to water. For the association on the current alcohol use and mothers’ sociodemographic factors, we used univariate and multivariate logistic regression analyses. The purposeful selection process began with a univariate analysis of each variable. Any variable having a significant univariate test at *p* < 0.20 was selected for the multivariate analysis. A stepwise backward elimination procedure was used to eliminate confounders. A probability level of 0.05 was considered significant.

### 2.7. Ethics Statement

This study adhered to the ethical principles and was approved by the Research and Ethics Committee of the Sefako Makgatho Health Sciences University (SMUREC) (SMUREC/H/169/2019: PG). Tshwane District Health Research Committee in Gauteng Province of South Africa granted us the permission to access the health facilities. Mothers gave consent to participate in the study with their newborns.

## 3. Results

### 3.1. Demography, Socio-Economy and Obstetric History of Mothers

The study population consisted of 300 child–mother pairs. The median age (IQR) of mothers was 29 years (24.0; 35.0). Most mothers were unemployed (63%), depended on child social grant (58.7%), and lived in households with a monthly income of ≤ $64.97 (41.3%). Some mothers reported that they used tobacco (18.7%) during pregnancy while very few used alcohol (3%). At the time of the study, 14.3% of mother were using tobacco while 49.7% were consuming alcohol. The prevalence of overweight (23.7%) and obesity (52%) among women were high, while underweight was low (3.3%) (Table 1).

Infant boys [178 (59.3%)] and girls [122 (40.6%)] with a mean age of 7.6 ± 3.2 months participated in the study with their mothers. Almost all mothers (93%) attended antenatal care, and at the time of the study, 202 (67.3%) mothers were breastfeeding (Table 2).

### 3.2. Malnutrition Indicators among Infants

WHO classification [53] was used to determine the prevalence of stunting (55%), underweight (41.7%), and thinness (22.3%) among infants. Although stunting and thinness affected both boys and girls equally, underweight was significantly different between boys and girls (*p* = 0.050) (Table 3).

### 3.3. Association of Tobacco and Alcohol Use with Underweight and Stunting

Table 4 shows the results of univariate and multivariable models for the association of tobacco and alcohol use with stunting, underweight, and thinness. Current tobacco use was associated with increased odds of being thin [OR = 2.40, 95% CI: 1.09–5.45), and after adjusting for confounders, current alcohol use was associated with the likelihood of being underweight [AOR = 1.96, 95% CI: 1.06–3.63] among infants. Analysis could not compute the association between current tobacco use and overweight, and between alcohol use during pregnancy for stunting, underweight and thinness due to multi-collinearity between these measures. Further multivariate analysis showed significant associations between current alcohol use with marital status [in a relationship, AOR = 2.28, 95% CI: 1.19–4.36, *p* = 0.013] and income (>R5000, AOR = 3.10, 95% CI: 1.69–5.68, *p* ≤ 0.0001). Underweight was associated with child’s sex [AOR = 0.55, 95% CI: 0.32–0.93, *p* = 0.027] and marital status [married; AOR = 0.27, 95% CI: 0.12–0.58, *p* = 0.001, and in a relationship; AOR = 0.45, 95% CI: 0.22–0.93, *p* = 0.031]. Thinness was associated with income [R1001–R5000; AOR = 2.8, 95% CI: 1.32–5.99, *p* = 0.007].

## 4. Discussion

The importance of maternal health and antenatal care in relation to infant growth and health cannot be over emphasised [41]. In the midst of a paucity of data, this study determined the relationship between maternal tobacco and alcohol use and malnutrition among infants. The study has been able to estimate tobacco and alcohol use among women of reproductive age during pregnancy and in the postnatal stage, which were associated with child malnutrition indicators in the context of a South African township setting. Additionally, associations of current maternal alcohol use and malnutrition indicators with socioeconomic factors were also reported in this study. A need to reduce alcohol use and smoking, and improve maternal and child nutrition, which consequently may promote child health and growth, requires evidence to contribute to interventions [41], and this study shed light on the phenomenon studied.

Mothers, together with their children, lived in unfavourable conditions in terms of large household sizes, unemployment rate, dependent on child social grant, and household monthly income of lesser or equal to $64.97. Pregnancy complications, such as miscarriage, premature delivery, stillbirth and death after birth, as well as child complications at birth, were observed in the study. The characteristics of a poor socioeconomic status and a poor obstetric history have been reported in several South African studies [41,44,47,56]. Optimal attendance of antenatal care among women in this study was comparable to the prevalence reported by the Department of Health, South Africa [1].

Considerable variations in tobacco use between the provinces and different racial groups in South Africa have been reported [16]. The current study was conducted in Gauteng Province and has reported the prevalence of tobacco use during pregnancy to be 18.7%. Over a decade ago, a study conducted in Gauteng Province reported 14.5% of pregnant women use tobacco [57]. As early as 2000, May et al. [17] reported high prevalence of tobacco use during pregnancy among mothers of children without FAS (46.3%) and mothers of children with FAS (87.9%) in the Western Cape Province. In addition, a recent prevalence of 36.8% has been reported among pregnant women who use substances in the Western Cape Province [58].

The prevalence of current use of tobacco among women in this study was 14.3%, which was higher compared to the finding reported in the South Africa Demographic and Health Survey (SADHS) (7%) [1] and the South African National Health and Nutrition Examination Survey (SANHANES-1) (7.3%) [16]. Western Cape (32.9%) has been reported as the province with the highest prevalence of current tobacco use, followed by Northern Cape (31.2%) and Free State (27.4%), while North West (12.7%) and Limpopo (12.8%) Provinces had the lowest prevalence [16]. Sociocultural and demographic determinants have been implicated on the use of tobacco [16]. Further results from SANHANES-1 showed that black Africans (15.1%) had a significant current tobacco use compared to the Coloureds (40.1%), Indians (22%), and Whites (15.3%) [16]. Although, not common in the current study, black South African women have been reported to use other types of tobacco, such as smokeless tobacco (14.6%) [11] and roll your own cigarette (15.8%) [10], both associated with poor socioeconomic characteristics [10,11], same as factors implicated with cigarette smoking [16].

Alcohol use among pregnant women in South Africa is a major concern, and regional varying rates have been observed [14]. The current study showed 3% of alcohol use among women during pregnancy, which is almost similar to a prevalence of 3.2% reported in the study of Erasmus conducted in Gauteng Province [57]. SANHANES-1, reported alcohol use among 3.7% of pregnant women, during 2011 and 2012 [19]. In the Western Cape Province, 42.8% of pregnant women reported drinking alcohol during pregnancy, and over half of them consumed enough alcohol to place their unborn children at high risk for FAS [17,18]. Further prevalence of alcohol use during pregnancy has been reported in Mpumalanga Province (6.5%) [59] and the Eastern Metropole District of Cape Town, in the Western Cape (20.4%) [58], while a relatively low prevalence has been reported in Limpopo Province (0.07%) [20].

Generally, South Africa has one of the highest levels of alcohol consumption, and this is mainly due to engagements on hazardous and harmful drinking [60]. The current study has reported a higher prevalence of current use of alcohol, which was estimated at 49.7%. The prevalence of the current use of alcohol in this study was higher than the prevalence in previous local surveys (16.9–17.1%) [15]. In addition, the SADHS has reported that 26% of South African women age 15+ using alcohol [1]. Literature has reported associations of alcohol use among women of reproductive age with the socioeconomic factors, like marital status, source of income, and employment status, among other factors [5,15]. Our study showed that the current use of alcohol was twice more likely among mothers who were in a relationship compared to those who were single, and three times likely among mothers with income more than $324.91 than those with an income of ≤$64.97.

Most women were breastfeeding (67.3%) at the time of the current study. A study conducted in the five communities of the Western Cape Province reported 71% of alcohol use among mothers of children with FASD during the postpartum period [27]. Not many local studies have reported the prevalence of maternal alcohol consumption while breastfeeding [27]. However, in high-income countries, a varying prevalence of alcohol use among women who were breastfeeding was reported in Australia (47%) [61], Canada (20%) [62], Netherlands (19%) [63], Norway (22%) [64], and United States (29% and 36%) [65]. Researchers have acknowledged that a substantial level of alcohol use during breastfeeding has noticeable effects on infants [66]. The possible mechanisms refer to the issue of doses of alcohol that might be delivered to the infant via breastmilk, making it impossible for infants to oxidize alcohol, as a result, affecting the development [25,67]. In their study, May et al. [27] concluded that despite the low amounts of alcohol passing from mother to a child, in addition to the difficulty to measure alcohol effects on children, breastmilk was found to be a significant enough factor to limit or delay physical growth and neurodevelopment of a child in the Western Cape Province.

Postnatal overweight (27.3%) and obesity (52%) among mothers were high in the current study, with minimal prevalence of underweight (3.3%). These findings are consistent with the reports of the SADHS, indicating high prevalence of overweight and obesity, and lower prevalence of undernutrition among women of reproductive age in Gauteng Province [1]. In the Western Cape Province, mothers of children with FASD were reported to be lighter and shorter with lower BMI [23]. Our findings show that mothers in Gauteng Province not only drink and smoke much less compared to mothers in the Western Cape, but they are much heavier, yet their infants are predisposed to underweight and thinness. This is a robust finding considering that mothers in the Western Cape Province are much thinner (i.e., lower BMI’s) and profoundly undernourished [23]. Literature documents that women with higher BMI have a greater amount of adipose tissue, which helps to distribute the alcohol, allowing less alcohol to cross the placenta [22]. Nonetheless, possibilities are that alcohol, as a teratogen, might suppress an infant’s development, even in mothers who are overweight and obese.

Stunting, underweight, and thinness were prevalent among infants in the current study, which was almost similar to previous studies [1,38,68,69]. Stunting among children reflects chronic undernutrition, while thinness shows the latest undernutrition status [54], and underweight combines stunting with wasting [70]. The current study showed that girls had lower odds of being underweight than boys, while infants whose mothers were married and in a relationship were less likely to be underweight, and infants whose mothers had income of between $65.04–$324.91 had increased odds of thinness. The association of malnutrition indicators with socioeconomic factors has been reported [41,44,45]. In contrast, concerning prevalence of overweight (8%) and obese (20.3%) among infants was reported in this study. There is minimal information on the prevalence of malnutrition indicators among infants of mothers who use tobacco and alcohol in South Africa. On the contrary, low prevalence of overweight (6%) and obesity (2%) among children have been reported in a cohort birth study conducted in South Africa [41]. Poor feeding practices and lifestyle modifications have been implicated in variation of the prevalence [71]. It is worth mentioning that the commonest health outcome of alcohol use on child development is FASD, ranging from 29 to 290 per 1000 live births in South Africa [72], representing the highest rate, globally, while the highest prevalence of FASD (between 196 and 276 children per 1000) in the country has been reported in the Western Cape Province [73].

The association of current alcohol use with the likelihood of being underweight was observed among infants in this study. Current tobacco use may predispose infants to thinness and growth problem, while tobacco use during pregnancy increased the odds of a growth problem (unadjusted model). Data on the association of tobacco and alcohol use during pregnancy and in the current period, with child malnutrition in South Africa, is scarce. However, one study reported that use of alcohol and tobacco during the antenatal period is a determinant of birthweight and growth in the first year of life [41]. In other countries, such as Indonesia [69] and Bangladesh [68], researchers have associated tobacco use with an increased risk of underweight and wasting (i.e., thinness). Although these studies further showed association with stunting, such was not the case in the current study. The authors linked the poorest households to these childhood issues, suggesting that the economic status could be a intermediating factor between smoking and child malnutrition [68,69].

Limited access to information on the use of tobacco and alcohol among women of reproductive age during the prenatal, antenatal, and postnatal periods condone use of these substances. Drinking during pregnancy is on the rise among women in several countries [74,75]. It is disturbing that most of the pregnancies are not planned and that could predispose the embryo to alcohol during early pregnancy [74,75]. Unplanned pregnancies constituted over a half of the total among women in this study, and the necessity to reduce unplanned pregnancies has been suggested by other researchers [5]. Lack of proper information on the possible health outcomes of alcohol use by potential mothers, and related fatal consequences, exacerbates drinking among women of reproductive age, leading to growth problems and morbidity and mortality among infants [14,41]. In the same way, tobacco use by mothers can be easily prevented from causing unfavourable pregnancy outcomes [76].

### Limitations

The current study had some limitations. Using a cross sectional design limited us to establish causality of the association. Retrospective assessment of maternal alcohol and tobacco use may have introduced recall bias. Even though the three questions used to measure the prevalence of tobacco use [77] were adopted in this study, alcohol assessment has always posed challenges due to self-reported recall, a frequently used method in epidemiological research [78]. Alcohol use might have been under estimated or under reported due to the social desirability of answering questions [79,80] and the dichotomous questions used. Use of a comprehensive tool that includes the quantitative measure, frequency, types, etc., to screen for alcohol use, such as the Alcohol Use Disorders Identification Test, should be considered in the future prospective studies. We acknowledge the limitation of not testing the reliability of the questionnaire statistically; however, the questionnaire was piloted before use. Since the study was conducted on average at 7 months postpartum period, data related to the time of pregnancy might also be at a high risk of recall bias. We further acknowledge that the study period for nutritional markers is very long (4 weeks–12 months). Probably it would be convenient if we could have adjusted the inclusion criteria to a shorter period or used a larger sample size than what we have used in the current study, in order to give greater relevance to the deviation of nutritional markers, which can be complicated given the targeted study population. Furthermore, during multivariate analysis, confounders were controlled for and our results are independent of these known confounders. Nonetheless, we could not eliminate the minimal remaining confounding completely. By itself, the study has too many confounding factors, which might influence the effect of alcohol on foetal development; as a result, a larger sample would be more appropriate. This could be the reason why association is not found in the adjusted model between alcohol and tobacco consumption, and stunting or thinness, despite these indicators being highly prevalent among children. Nonetheless, this study provides the basis for focusing interest on this important topic and promoting local prospective studies with a larger population.

## 5. Conclusions

This study showed the use of tobacco and alcohol during pregnancy, and mothers continuing to smoke and drink in the postnatal period (i.e., current period). Current alcohol use was associated with marital status and income. The prevalence of malnutrition indicators was high, especially for stunting and underweight. The co-occurrence of malnutrition indicators with tobacco and alcohol use pose some growth threats among infants. Significant associations were observed between current alcohol use and the likelihood of being underweight, while current tobacco use predisposed infants to thinness. Alcohol use by women who were breastfeeding appeared to be relatively common; however, the relationship surrounding alcohol use and breastfeeding could be difficult to determine. Given the status of tobacco and alcohol use among women of reproductive age and the high prevalence of child malnutrition, programmes to protect children from being exposed to maternal use of the above-mentioned substances from the intrauterine life to infancy are imperative. Furthermore, it is important that health care providers give convincing counselling on approaches to avoid tobacco and alcohol use, especially during the antenatal and postnatal care. Future prospective cohort studies examining malnutrition among infants exposed to maternal tobacco and alcohol use from birth to at least 6 months are necessary to inform, partly, the public health strategies aimed at decreasing child malnutrition. There is a need for an analytical approach and rigorous application of standardized methods to assess tobacco and alcohol use in relation to child malnutrition. Because malnutrition is multifactorial, there is also a need to consider and control for other determinants during such an analytical approach.

## Figures and Tables

**Table 1:** Characteristics of mothers (*n* = 300).

Variables	Categories	*n*	%
Age (years)	≤30	176	58.7
	>30	124	41.3
BMI (kg/m^2^)	Normal	63	21
	Underweight	10	3.3
	Overweight	71	23.7
	Obesity	156	52
Marital status	Single	56	18.7
	Ever married	100	33.3
	In a relationship	144	48.0
Employment	No	189	63.0
	Yes	111	37.0
Education	No school/primary	2	0.7
	Secondary	72	24.0
	Completed and post grade 12	226	75.3
Receiving child social grant	No	124	41.3
	Yes	176	58.7
Household size	<4	188	62.7
	≥5	112	37.3
House type	RDP	29	9.7
	Brick	240	80.0
	Shack	31	10.3
Household income	≤$64.97	124	41.3
	$65.04–$324.91	95	31.7
	$324.91	81	27.0
Water access	No	7	2.3
	Yes	293	97.7
Electricity	No	12	4.0
	Yes	288	96.0
Refrigerator use	No	16	5.3
	Yes	284	94.7
Tobacco use during pregnancy	No	245	81.7
	Yes	55	18.3
Alcohol use during pregnancy	No	292	97.0
	Yes	8	3.0
Current tobacco use	No	257	85.7
	Yes	43	14.3
Current alcohol use	No	151	50.3
	Yes	149	49.7

BMI stands for body mass index, RDP = Reconstruction and Development Programme house, *n* indicates frequency, and % indicates percentage.

**Table 2:** Obstetric history of mothers and infants (*n* = 300).

Variables	Categories	*n*	%
Parity	1–2	86	28.7
	≥3	214	71.3
Child gender	Boys	178	59.3
	Girls	122	40.7
Pregnancy planned	No	175	58.3
	Yes	125	41.7
Attended ANC	No	20	6.7
	Yes	280	93.3
ANC initiation ^#^	Less than 1 month	87	31.1
	2–3 months	105	35.0
	Over 3 months	88	29.3
Pregnancy term	Full	245	81.7
	Premature	55	18.3
Infant birth weight	<2.5 kg	1	0.3
	≥2.5 kg	299	99.7
Pregnancy complications	No	250	83.3
	Yes	50	16.7
Currently breastfeeding	No	98	32.7
	Yes	202	67.3
Diagnosed with a disease during pregnancy	No	277	92.3
	Yes	33	7.7
Discharged same day after delivery	Yes	137	45.6
	No	163	54.3
Child complications at birth	Yes	60	20
	No	240	80

ANC stands for antenatal care, *n* indicates frequency, % indicates percentage and # indicates *n* = 280.

**Table 3:** Comparison of medians of anthropometry and malnutrition indicators in infants by sex.

Variables	All	Boys	Girls	*p*-Value
	*n*= 300	*n*= 178	*n*= 122	
Weight (kg)	6.9 (5.2; 91)	6.9 (5.1; 9.0)	6.8 (5.2; 9.1)	0.899
Height (cm)	60 (51; 70)	60 (50; 70)	60 (54; 70)	0.926
HAZ—median (IQR)	−2.62 (−6.24; 0.98)	−3.26 (−6.68; 0.28)	−1.81 (−5.44; 1.87)	0.090
Normal—*n* (%)	60 (20.0)	33 (18.5)	27 (22.1)	
Stunting—*n* (%)	165 (55.0)	105 (58.9)	60 (49.2)	0.847
Tallness—*n* (%)	75 (25.0)	40 (22.5)	35 (28.7)	0.239
WAZ—median (IQR)	1.48 (−3.02; 0.32)	−1.87 (−3.52; 0.08)	−1.12 (−2.47; 0.61)	0.007 *
Normal—*n* (%)	127 (42.5)	69 (38.8)	58 (47.5)	
Underweight—*n* (%)	125 (41.7)	83 (46.6)	42 (34.4)	0.050 *
Growth problem—*n* (%)	48 (16)	26 (14.6)	22 (18)	0.984
BAZ—median (IQR)	0.11 (−1.78; 2.29)	0.03 (−1.89; 2.23)	0.63 (−1.65; 2.44)	0.436
Normal	114 (38)	68 (38.2)	46 (37.7)	
Thinness	67 (22.3)	42 (23.6)	25 (20.5)	0.686
Overweight risk	34 (11.3)	17 (9.6)	17 (13.9)	0.318
Overweight	24 (8)	16 (9.0)	8 (6.7)	0.552
Obesity	61 (20.3)	35 (19.7)	26 (21.3)	0.771

IQR stands for interquartile range, HAZ = height for age Z-score, WAZ = height for age Z-score, stunting is defined as HAZ < −2 SD, underweight defined as WAZ < −2 SD and BAZ defines thinness, overweight and obesity. * indicates significant difference.

**Table 4:** Univariate and multivariate association between tobacco and alcohol use and malnutrition indicators.

Variables	Univariate ^a^		Multivariate ^b^	
**Current Period**				
***Tobacco Use***	**OR (95% CI)**	***p* Value**	**AOR (95% CI)**	***p* Value**
Stunting	0.80 (0.33–193)	0.616	0.67 (0.23–1.93)	0.455
Tallness	1.91 (0.76–4.78)	0.170	1.93 (0.67–5.89)	0.244
Underweight	1.83 (0.85–3.91)	0.122	2.44 (1.00–6.18)	0.060
Growth problem	2.85 (1.17–6.99)	0.022 *	1.60 (0.55–4.64)	0.383
Thinness	2.40 (1.09–5.45)	0.030 *	1.72 (0.70–4.21)	0.240
Overweight risk	0.75 (0.20–2.81)	0.672	0.83 (0.22–3.21)	0.792
Obesity	1.71 (0.71–4.09)	0.228	2.49 (0.93–6.63)	0.069
***Alcohol use***				
Stunting	1.00 (0.55–1.81)	1.000	0.72 (0.35–1.46)	0.362
Tallness	1.62 (0.82–3.22)	0.166	1.98 (0.87–4.54)	0.102
Underweight	1.42 (0.86–2.33)	0.167	1.96 (1.05–3.63)	0.033 *
Growth problem	1.63 (0.83–3.18)	0.152	0.96 (0.42–2.21)	0.926
Thinness	1.28 (0.70–2.34)	0.428	0.91 (0.46–1.78)	0.776
Overweight risk	0.64 (0.29–1.40)	0.266	0.74 (0.33–1.66)	0.464
Overweight	0.62 (0.25–1.54)	0.302	0.87 (0.33–2.26)	0.768
Obesity	1.30 (0.70–2.44)	0.405	1.86 (0.90–3.86)	0.092
**Pregnancy Period**				
***Tobacco Use***	**OR (95% CI)**	***p* Value**	**AOR (95% CI)**	***p* Value**
Stunting	0.72 (0.33–1.59)	0.417	0.70 (0.27–1.78)	0.449
Tallness	1.73 (0.76–3.96)	0.192	1.55 (0.57–4.19)	0.391
Underweight	1.54 (0.78–3.03)	0.213	1.85 (0.80–4.25)	0.150
Growth problem	2.66 (1.19–5.96)	0.017 *	1.71 (0.65–4.50)	0.276
Thinness	1.63 (0.79–3.34)	0.184	1.25 (0.56–2.79)	0.593
Overweight risk	0.59 (0.19–186)	0.367	0.63 (0.20–2.05)	0.447
Overweight	0.19 (0.02–1.51)	0.117	0.27 (0.04–2.2.1)	0.224
Obesity	0.97 (0.43 −2.18)	0.950	1.28 (0.52–3.18)	0.593

OR stands for odd ratio, AOR for adjusted odds ratio, CI for confidence interval, and * indicates a significant association at *p* < 0.05. ^a^ Estimated with logistic regression model for categorical variables and ^b^ adjusting for child’s sex, maternal age, BMI, marital status, education level, employment status, family size, monthly income, house type, access to water, parity and pregnancy complications.

## Data Availability

The dataset for children and their mothers generated and analysed during the current study are available from the corresponding author upon reasonable request.
